# Supplementary figures and images for: Graph topological transformations in space-filling cell aggregates
Source: PLoS Comput Biol. 2024 May 14;20(5):e1012089. doi: 10.1371/journal.pcbi.1012089 (PMC11093388; doi:10.1371/journal.pcbi.1012089)

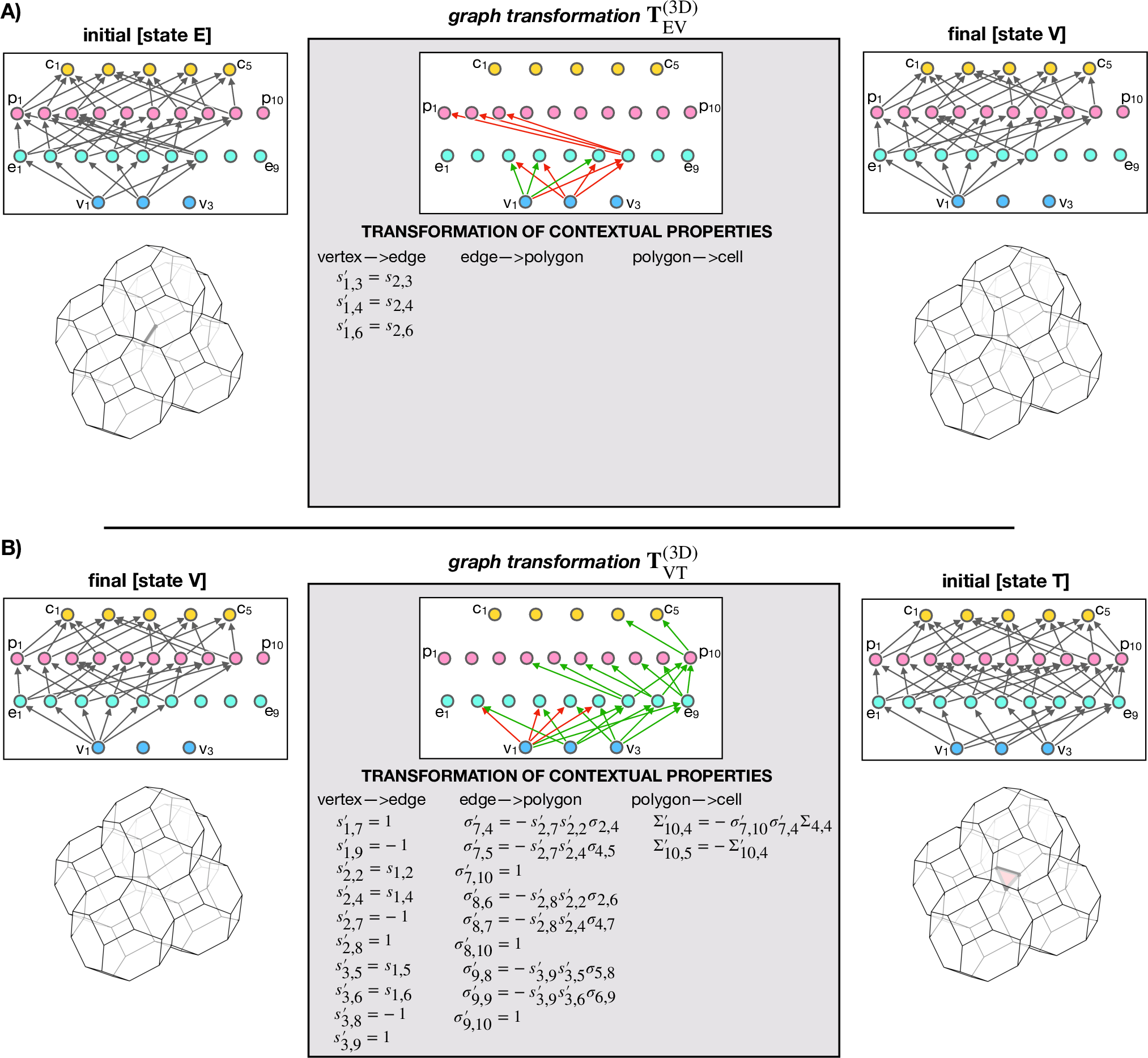

Supplement: S1 Fig — (panels A and B, respectively). Graphs in the left and the right column correspond to the initial and the final cell configuration, respectively. Gray arrows represent relationships labeled IS_PART_OF. The graphs in the middle column show graph transformations, which include green and red relationships, indicating relationship creations and deletions, respectively. Additionally, the graph transformation specifies property values of the newly created relationships. In each graph, the node indices increase from left to right in unit steps. (TIF) [file pcbi.1012089.s001.tif]

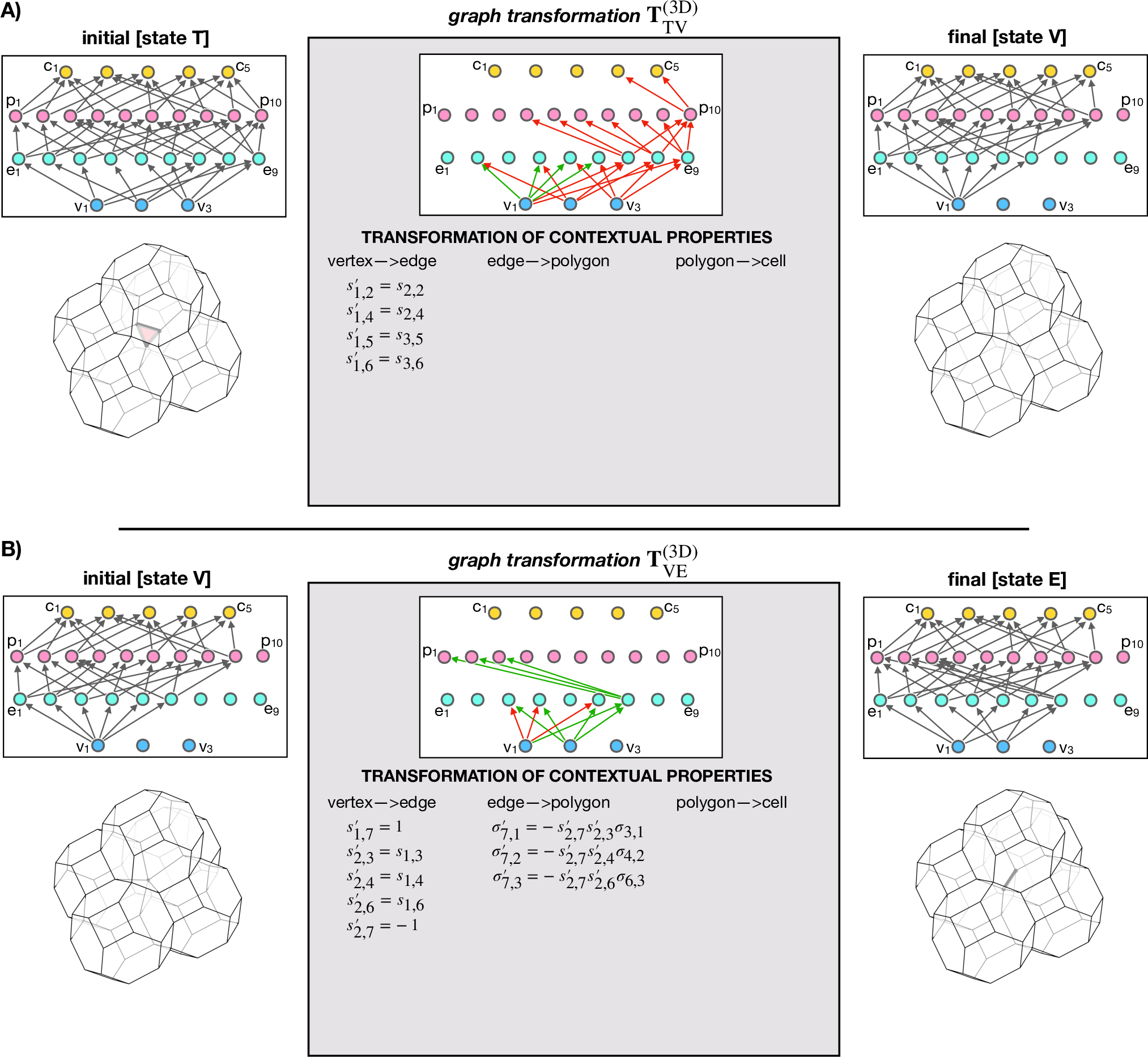

Supplement: S2 Fig — (panels A and B, respectively). Graphs in the left and the right column correspond to the initial and the final cell configuration, respectively. Gray arrows represent relationships labeled IS_PART_OF. The graphs in the middle column show graph transformations, which include green and red relationships, indicating relationship creations and deletions, respectively. Additionally, the graph transformation specifies property values of the newly created relationships. In each graph, the node indices increase from left to right in unit steps. (TIF) [file pcbi.1012089.s002.tif]

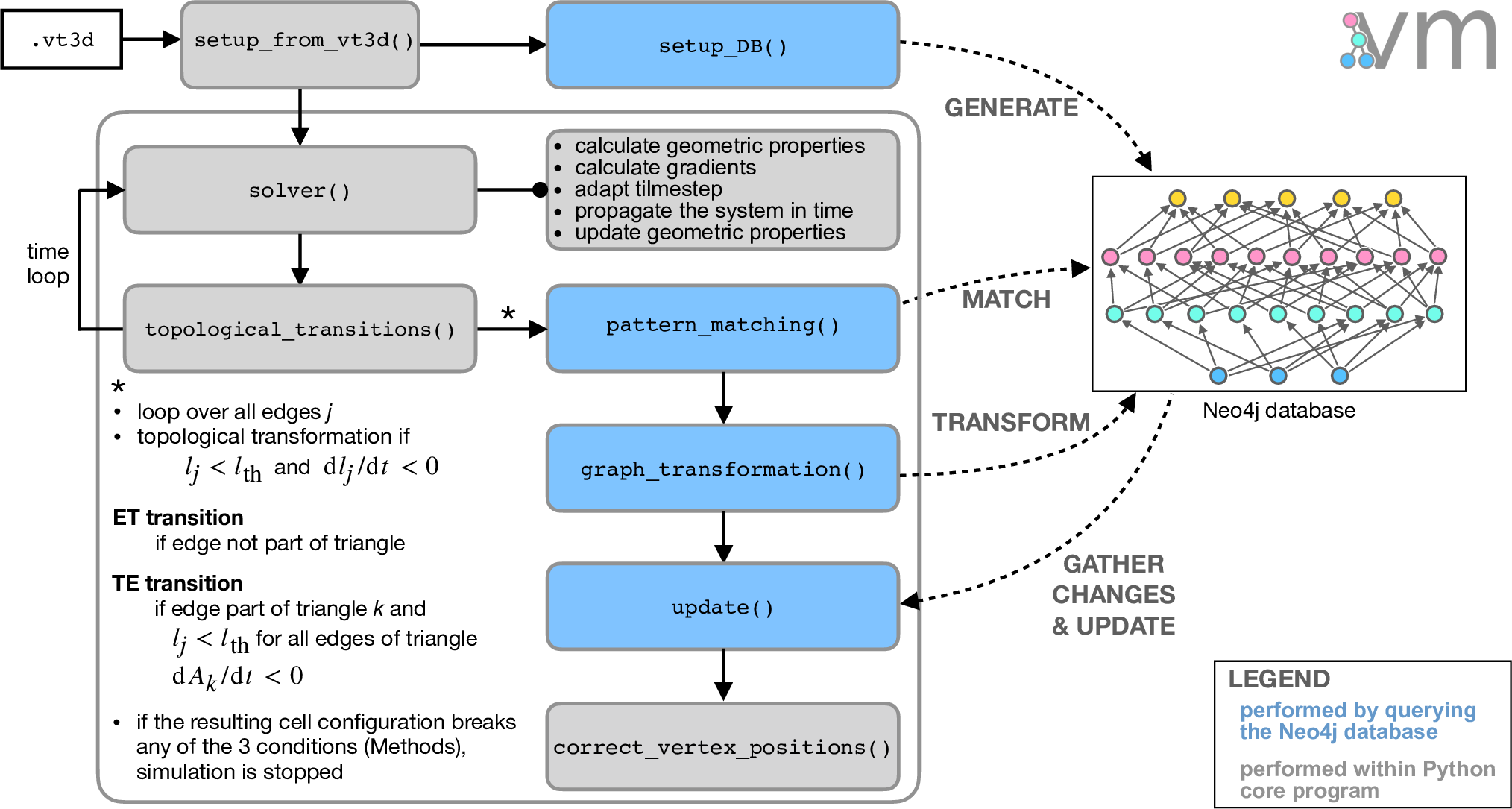

Supplement: S3 Fig — A 3D cell aggregate is set up from an input .vt3d file by function setup_from_vt3d() and then converted into a graph database, set up in Neo4j by function setupDB(). These initialization steps then followed by a time loop, which iterates between solver() and topological_transitions() functions. The function solver() calculates geometric properties of cells and the associated gradients (i.e., conservative forces) and propagates the system forward in time. The function topological_transitions() loops over all cell edges to find those that meet criteria for topological transitions. For edges that meet these criteria, topological transitions are performed through pattern matching and graph transformations (functions pattern_matching() and graph_transformation(), respectively), applied directly to the graph database. Finally, the tissue is updated accordingly by function update() and positions of vertices involved in the topological transformation are corrected by function correct_vertex_positions(). (TIF) [file pcbi.1012089.s003.tif]
